# Alzheimer Disease and Selected Risk Factors Disrupt a Co-regulation of Monoamine Oxidase-A/B in the Hippocampus, but Not in the Cortex

**DOI:** 10.3389/fnins.2018.00419

**Published:** 2018-06-26

**Authors:** Maa O. Quartey, Jennifer N. K. Nyarko, Paul R. Pennington, Ryan M. Heistad, Paula C. Klassen, Glen B. Baker, Darrell D. Mousseau

**Affiliations:** ^1^Cell Signalling Laboratory, Department of Psychiatry, University of Saskatchewan, Saskatoon, SK, Canada; ^2^The Pharmacology-Physiology Honours Program, University of Saskatchewan, Saskatoon, SK, Canada; ^3^Neurochemical Research Unit, Department of Psychiatry, University of Alberta, Edmonton, AB, Canada

**Keywords:** Alzheimer disease, depression, monoamine oxidase, APOE4, sex/gender risk

## Abstract

Monoamine oxidase-A (MAO-A) and MAO-B have both been implicated in the pathology of Alzheimer disease (AD). We examined 60 autopsied control and AD donor brain samples to determine how well MAO function aligned with two major risk factors for AD, namely sex and *APOE* ε4 status. MAO-A activity was increased in AD cortical, but not hippocampal, samples. In contrast, MAO-B activity was increased in both regions (with a strong input from female donors) whether sample means were compared based on: (a) diagnosis alone; (b) diagnosis-by-*APOE* ε4 status (i.e., carriers vs. non-carriers of the ε4 allele); or (c) *APOE* ε4 status alone (i.e., ignoring ‘diagnosis’ as a variable). Sample means strictly based on the donor’s sex did not reveal any difference in either MAO-A or MAO-B activity. Unexpectedly, we found that cortical MAO-A and MAO-B activities were highly correlated in both males and females (if focussing strictly on the donor’s sex), while in the hippocampus, any correlation was lost in female samples. Stratifying for sex-by-*APOE* ε4 status revealed a strong correlation between cortical MAO-A and MAO-B activities in both non-carriers and carriers of the allele, but any correlation in hippocampal samples was lost in carriers of the allele. A diagnosis of AD disrupted the correlation between MAO-A and MAO-B activities in the hippocampus, but not the cortex. We observed a novel region-dependent co-regulation of *MAO-A* and *MAO-B* mRNAs (but not proteins), while a lack of correlation between MAO activities and the respective proteins corroborated previous reports. Overexpression of human APOE4 increased MAO activity (but not mRNA/protein) in C6 and in HT-22 cell cultures. We identified a novel co-regulation of MAO-A and MAO-B activities that is spared from any influence of risk factors for AD or AD itself in the cortex, but vulnerable to these same factors in the hippocampus. Sex- and region-dependent abilities to buffer influences on brain MAO activities could have significant bearing on ambiguous outcomes when monoaminergic systems are targeted in clinical populations.

## Introduction

The two isoforms of monoamine oxidase [amine: oxygen oxidoreductase (deaminating) (flavin containing), EC 1.4.3.4, monoamine oxidase (MAO)], i.e., MAO-A and MAO-B, are expressed primarily on the mitochondria. Altered function of either isoform or any associated disruptions in the degradation of biogenic amine neurotransmitter substrates such as serotonin, dopamine, and noradrenaline, have been associated with disorders as varied as depression, cancers, and neurodegeneration (e.g., Parkinson’s disease and Alzheimer disease/AD) (Mousseau and Baker, 2012). MAO may also be a factor in neuropathology because of the generation of hydrogen peroxide as a by-product of the deamination reaction. The ensuing oxidative stress and potential for cell death –invariably involving the mitochondria– would be exacerbated when antioxidant systems are compromised, such as during aging (Zhu et al., 2006) and particularly in AD (Crack et al., 2006).

The loss of MAO-A-immunoreactive cells is exacerbated in brainstem monoaminergic nuclei and other regions in late-stage cognitive decline (Chan-Palay et al., 1993). MAO-B is primarily expressed in glia (Riederer et al., 1987) and its role in neurodegeneration has also been widely studied (Inaba-Hasegawa et al., 2017; Riederer and Muller, 2017). MAO-A/-B-associated change in aminergic neurotransmitter levels in AD (Nazarali and Reynolds, 1992; Sjogren et al., 1998; Lai et al., 2002) likely contributes to the neurobiology of a range of neuropsychiatric symptoms observed in AD populations (Li et al., 2014; Vermeiren et al., 2014; Rosenberg et al., 2015). For example, depression, which has been historically associated with monoaminergic dysfunction, has been proposed to represent a prodrome for AD-related dementia in certain vulnerable cohorts (Geerlings et al., 2008; Caraci et al., 2010; Wuwongse et al., 2010), while anxiety and aggression in individuals with mild cognitive impairment might indicate imminent conversion to AD (Gallagher et al., 2011). Furthermore, changes in levels of the MAO-mediated acid metabolites of serotonin and dopamine – i.e., 5-hydroxyindole-3-acetic acid (5-HIAA) and homovanillic acid (HVA), respectively– have long been associated with cognitive deficits and dementia (Gottfries et al., 1969a,b; Nazarali and Reynolds, 1992; Vermeiren et al., 2015) and are observed in diverse mouse models of AD-related pathology (Ash et al., 2010; Wei et al., 2012). The effect of monoamines in AD might extend beyond a contribution to neuropsychiatric symptoms; for example, the cleavage of the Amyloid Protein Precursor (APP: which yields the toxic β-amyloid (Aβ) peptide in AD) is sensitive to 5-HT *via* activation of the 5-HT2a, 5-HT2c, and 5-HT4 receptors (Nitsch et al., 1996; Cochet et al., 2013). In addition, levels of monoamine acid metabolites have been positively correlated with cerebrospinal levels of Aβ (Stuerenburg et al., 2004), while MAO-B-positive astrocytes are detected in the vicinity of amyloid plaques, a hallmark of AD neuropathology (Saura et al., 1994). This latter association has been re-confirmed recently using two-photon imaging in the 5xFAD mouse model of AD (Kim et al., 2016). Reversible inhibitors of MAO-A, such as moclobemide, have shown modest results in elderly individuals, including those presenting with cognitive deficits (Rosenzweig et al., 1998; Gareri et al., 2000), whereas inhibitors of MAO-B, such as l-deprenyl, might provide benefit in the early stages of clinical neurodegenerative diseases, such as Parkinson’s disease (Magyar et al., 2004; Youdim et al., 2006) and mild AD-type dementia (Riederer et al., 2004).

Female sex is a risk for AD and estrogen has been shown to reduce *MAO-A* mRNA in the macaque dorsal raphé (Gundlah et al., 2002) and several regions of the rat brain (Holschneider et al., 1998), while progesterone affects platelet MAO (i.e., MAO-B) activity (Klaiber et al., 1996). Regional brain MAO-B activity is increased in AD (Adolfsson et al., 1980; Oreland and Gottfries, 1986) and mean platelet MAO-B activity is increased in female AD patients (Robinson et al., 1971; Veral et al., 1997), yet it is not clear how much of this change might rely on the patient’s biological sex or on other factors such as the widely-acknowledged genetic risk for late-onset AD, i.e., the *APOE* ε4 allele (Poirier et al., 1993). A single ε4 allele can impart significant risk in women, with little effect in men (Payami et al., 1996; Farrer et al., 1997; Bretsky et al., 1999). Furthermore, female carriers (but not males) might be more likely to have been depressed *prior* to developing AD (Delano-Wood et al., 2008) with the interaction between ε4 genotype and depression increasing the risk of incident dementia (Meng and D’Arcy, 2013). Although a larger cohort study has not supported the *APOE* ε4 allele as a risk factor for depression as a prodrome in AD (Locke et al., 2013), another study has found an interaction between *APOE* ε4 genotype and depression, as well as a higher risk of incident mild cognitive impairment in male depressed patients (Geda et al., 2006). Despite evidence such as this, most clinical research views male and female *APOE* ε4 carriers as having equal risk (Altmann et al., 2014) and persist in pooling data from males and females, which has likely biased outcomes and any extrapolation of data generated in these contexts.

The fact that monoaminergic systems are affected in early stages of AD is clear. What remains unclear is whether monoaminergic function is more closely aligned with risk or with diagnosis of AD. Previous studies have associated the *APOE* ε4 allele with decreased *mao-A* mRNA expression in C6 glioblastoma cells (Liu et al., 2012), while risk in a Brazilian cohort of 128 late-onset AD patients was associated with combined *MAO-A* polymorphism (allele 1; lower transcription efficiency), the short variant of the serotonin transporter promoter, and a positive *APOE* ε4 status (Nishimura et al., 2005). Our work differs from previous reports in that we used several stratification approaches that would allow us to draw some conclusions as to the degree to which factors such as brain region, sex, and *APOE* ε4 status could be influencing any AD-related, MAO-associated neurochemical phenotype. Our results strongly align changes in MAO (particularly MAO-B) function with a diagnosis of AD and with the *APOE* ε4 risk factor. As importantly, or perhaps more so, a closer examination of the data suggests that MAO-A and MAO-B activities in the cortex are tightly co-regulated and that this co-regulation is not overtly affected by the donor’s sex or *APOE* ε4 status, or by a diagnosis of AD. This is in contrast to the hippocampus where the co-regulation is far less robust in the female and where this co-regulation appears to be vulnerable in carriers of the *APOE* ε4 allele and in individuals with a diagnosis of AD. This could explain some of the region-dependent pathology associated with AD and, possibly, some of the variable response to MAO inhibitors in clinical trials.

## Materials and Methods

### Human Brain Samples

Sixty cortical samples were obtained from the Douglas–Bell Canada Brain Bank (McGill University, Montréal, QC, Canada). These included 16 male and female (M/F) early-onset AD (EOAD: 7M/9F), 18 late-onset/sporadic AD (LOAD: 8M/10F), and 26 controls (CTL: 12M/14F) matched as closely as possible for age and sex (see section “*Donor Statistics*,” Supplementary Table 1). Cortical samples were randomly chosen from left and right hemispheres, and represent a mix of superior and middle frontal cortices (Brodmann Areas 9/46, respectively). These areas are associated with executive function and cognition, and are a target of relative hypoperfusion in AD patients, particularly those with a co-morbid depression (Levy-Cooperman et al., 2008). Diagnoses were histopathologically confirmed by on-site pathologists (using CERAD criteria). Wherever available, we examined the corresponding hippocampal samples from each donor. Our 60-sample sets were randomly assigned at source, without any information available regarding the donors’ *APOE* ε4 status.

De-identified samples were assayed under the University of Saskatchewan’s Research Ethics Office Certificate of Approval ‘Bio 06-124’ (held by DM).

### Reagents and Antibodies

The anti-GAPDH antibody (14C10: #2118) was purchased from Cell Signaling Technology (Whitby, ON, Canada). [2-^14^C]-5-HT binoxalate ([^14^C]-5-HT: NEC-225) and β-[ethyl-1-^14^C]-PEA hydrochloride ([^14^C]-PEA: NEC-502) were purchased from PerkinElmer Life Sciences (Waltham, MA, United States). The MAO-A (H-70) and MAO-B (C-17) antibodies were purchased from Santa Cruz Biotechnology (Santa Cruz, CA, United States). The anti-Apolipoprotein E antibody (D6E10; ab1906) was purchased from Abcam. IgG-HRP conjugates were from Bio-Rad Laboratories Ltd. (Mississauga, ON, Canada). All other reagents were obtained from commercial sources.

### Monoamine Oxidase (MAO) Catalytic Activity

MAO-A and MAO-B catalytic activities were estimated using 250 μM [^14^C]-5-HT and 50 μM [^14^C]-PEA, respectively, and 100 μg total cell protein *per* reaction in oxygenated potassium phosphate buffer (0.2 M, pH 7.8) (Cao et al., 2009b). The 10-min reaction was terminated by addition of 25 μL of HCl, after which the radiolabeled reaction products were extracted into 1 mL of water-saturated ethyl acetate/toluene. Samples were centrifuged and 700 μL of the organic phase was used to determine radioactive content using scintillation spectrometry. Individual sample means represent the average of 3–5 replicates.

### Immunodetection

Standard SDS-PAGE denaturing conditions were used to detect expression of target proteins in cleared (12,000 ×*g*, 10 min, 4°C) lysates (15 μg/lane) (Cao et al., 2009b; Wei et al., 2012). Detection relied on enhanced chemiluminescence and ImageJ 1.32j^1^ was used for densitometric analyses of scanned blots.

### Quantitative Real-Time PCR

Total RNA was isolated using an RNeasy^®^ Mini Kit (Qiagen; Mississauga, ON, Canada) and reverse-transcribed to cDNA using iScript Select cDNA Synthesis Kit from Bio-Rad (Cat # 170-8897). Gene expression was quantified using Taqman^®^ primers, specifically (Hs00165140_m1, spans Ex6-7 boundary of *MAO-A*) and (Hs01106246_m1, spans Ex2-3 boundary of *MAO-B*) from Applied Biosystems (Foster City, CA, United States). Triplicate reactions were performed using the Taqman Universal Master Mix, FAM-labeled Taqman Gene Expression assays for the target gene, VIC-labeled Taqman Endogenous Control GAPDH, and 500 ng of cDNA and thermocycling parameters as described previously (Wei et al., 2012).

### High Pressure Liquid Chromatography (HPLC)

Levels of MAO-mediated acid metabolites of serotonin and dopamine, i.e., 5-HIAA and HVA, respectively, were determined by HPLC with electrochemical detection as described before and based on comparing peak heights of the analytes to those of a set of authentic standards processed in parallel (Wei et al., 2012). Note that HVA, rather than DOPAC, is the primary acid metabolite of dopamine in the human brain (Ebinger et al., 1987; Vermeiren et al., 2015). In addition, levels of DOPAC were not consistently detected across samples. As such DOPAC was excluded from any analyses.

### APOE Genotyping

The APOE2, APOE3, and APOE4 variants differ by arginine (Arg) and/or cysteine (Cys) substitutions, i.e., Cys/Cys (E2), Cys/Arg (E3), and Arg/Arg (E4), at positions 112 and 158, respectively. *APOE* restriction isotyping for the two single nucleotide polymorphisms encoding for these substitutions was done as described fully elsewhere (Nyarko et al., 2018). Briefly, PCR amplification was performed on 500 ng of genomic DNA and the resulting 226 bp amplicon was restricted with *Afl*III and *Hae*II. The fragments were resolved on a 10% non-denaturing, polyacrylamide gel and visualized by staining with GelRed (Biotium). Genotyping identified ε2, ε3, and ε4 homo- and heterozygotes, with the frequency of ε4 carriers across cases in our sample set supporting those reported in the literature (Poirier et al., 1993).

### Expression of Human APOE Variants in Immortalized Neuronal and Glial Cell Cultures

Human *APOE* cDNA was cloned from an *APOE* ε3/ε3 donor sample as well as from an *APOE* ε4/ε4 donor sample. Primers used were: sense 5′-*Hind*III-APOE: tgc aag ctt atg aag gtt ctg tgg gct gcg ttg; and antisense 5′-*BamH*I-APOE: agt gga tcc tca gtg att gtc gct ggg cac ag. The fragments were subcloned into pcDNA3.1/Hygro(+) and confirmed by sequencing. The *APOE* ε3 cDNA was used to generate an Arg158Cys substitution by mutating the cgc (Arg) codon to a ***tgc*** (Cys) codon using the sense-5′ primer: at gac ctg cag aag ***tgc*** ctg gca gtg tac cag, and complementary primer: tg gta cac tgc cag ***gca*** ctt ctg cag gtc atc. The resulting *APOE* ε2 cDNA was confirmed by sequencing.

Rat C6 glioblastoma cells (ATCC: CCL-107) and mouse immortalized HT-22 hippocampal cells (Maher and Davis, 1996) were transfected with the individual *APOE* ε2, ε3, or ε4 expression plasmids. Cell pellets were collected 24 h later for mao-A and mao-B catalytic activity assays, for Western blotting, and for *mao-A* and *mao-B* mRNA levels.

### Statistical Analyses

Any possibility of bias using our autopsy-derived data was minimized by having some individuals assay de-identified samples and others perform the analysis. For example, for Western blotting, one individual prepares, resolves, and probes the protein blot, another individual scans the blot and performs the densitometric analysis, and another individual analyzes the data. This, in principle, effectively dilutes the possibility of any single observer bias. Data were analyzed using either the Mann–Whitney *U* test or ANOVA (Kruskal–Wallis) with *post hoc* multiple comparisons using Dunn’s test. Significance was set at *P*< 0.05, but values that fell between 0.051 and 0.10 were discussed as *tendencies*. Only those *P*-values associated with a significant change are included (*note*, a non-significant *P*-value might be included to support a pivotal conclusion). Data are represented as scatter plots with the lines representing the sampling mean ± standard deviation. Correlation statistics were based on Pearson’s correlation coefficient (*r*). Note that we will use the term ‘correlated’ to mean ‘positively correlated,’ unless otherwise stated. We acknowledge that a limitation in the interpretation of our data is that our 60-sample set was not sufficiently powered to undertake statistically relevant three-way stratification, i.e., sex-by-diagnosis-by-genotype.

## Results

This report identifies changes in monoaminergic profiles that align strongly with the donor’s sex and *APOE* ε4 status (i.e., as carrier, or not, of an ε4 allele), and also identifies a regionally dependent co-regulation of MAO-A and MAO-B function. In the cortex, this co-regulation is spared from any influence of risk factors for AD, such as sex or the *APOE* ε4 allele. In the hippocampus, this co-regulation is disrupted by these same risk factors.

### Donor Statistics

De-identified information on the basic metrics of the donors – i.e., sex and age at time of death, and the post-mortem interval and brain weight– are summarized in Supplementary Table 1. Briefly, there was the expected difference in the age of the donors based on diagnosis (control: 70.7 ± 12.5; EOAD: 58.1 ± 7.8, *P* < 0.05; LOAD: 83.2 ± 5.8, *P* < 0.01). There was a difference in average brain weight (g) based on diagnosis (control: 1232 ± 137; EOAD: 1016 ± 200, *P* < 0.01; LOAD: 1036 ± 122, *P* < 0.001). There was no difference in post-mortem interval between the groups.

In general, we did not observe any significant correlation between the activity of either isoform in cortical samples and (a) post-mortem interval, (b) age at time of death, or (c) brain weight. In contrast, we did observe a few significant correlations in the hippocampal sample sets. These included a negative correlation (*P* = 0.0021) between MAO-A activity and age at time of death in the LOAD sample set; a positive correlation (*P* = 0.0053) between MAO-A activity and brain weight in the control sample set; and a negative correlation (*P* = 0.0160) between MAO-B activity and brain weight in the EOAD sample set.

### MAO-A and MAO-B Parameters Based on the Donor’s Diagnosis

**FIGURE 1:**
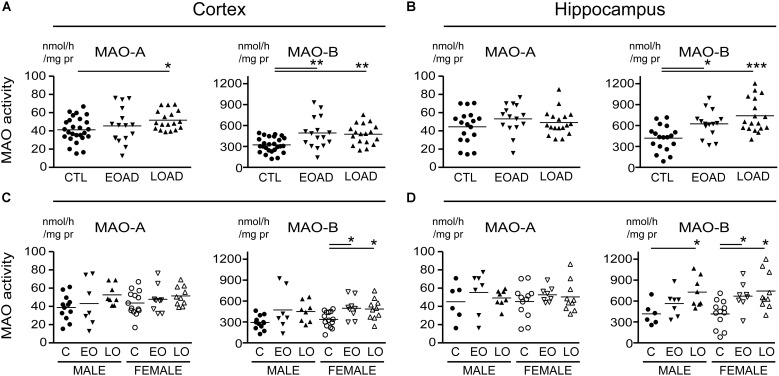
Monoamine oxidase (MAO) activites in human brain: data analyzed based on the donor’s diagnosis. MAO-A and MAO-B activites were measured in **(A)** the cortex and **(B)** the hippocampus of control (CTL, C), Early-Onset AD (EOAD, EO), and Late-Onset AD (LOAD, LO) samples by radioenzymatic assay. **(C,D)** The same samples re-analyzed based on the donor’s sex. ^∗^*P* < 0.05; ^∗∗^*P* < 0.01; ^∗∗∗^*P* < 0.001 between indicated groups.

**FIGURE 2:**
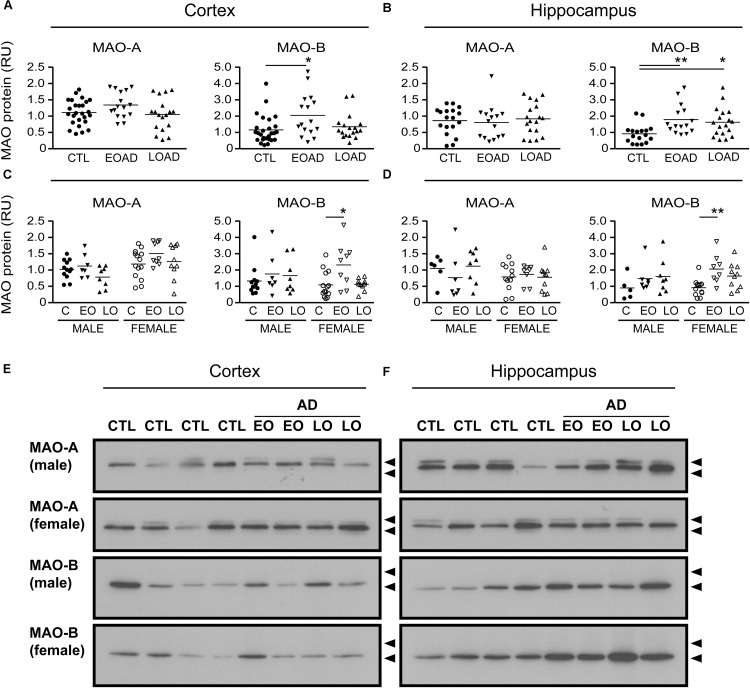
MAO proteins in human brain: data analyzed by diagnosis. Western blots were probed for MAO-A and MAO-B proteins. Scans were analyzed by densitometry in **(A)** the cortex and **(B)** the hippocampus of control (CTL, C), Early-Onset AD (EOAD, EO), and Late-Onset AD (LOAD, LO) samples. **(C,D)** The samples were separated based on the donor’s sex. ^∗^*P* < 0.05; ^∗∗^*P* < 0.01 between indicated groups. **(E,F)** Representative immunoblots show the MAO-A and MAO-B protein bands in **(E)** cortical and **(F)** hippocampal extracts from male and female donors. Lower arrowhead (55 kDa) and upper arrowhead (72 kDa) represent protein standard markers.

MAO-A activity was increased in cortical AD samples (*P* = 0.0528), but not in hippocampal AD samples (*P* = 0.3614). In contrast, MAO-B activity was increased in AD samples in both cortex (*P* = 0.0009) and hippocampus (*P* = 0.0005), with contributions from both sexes (cortex: males, *P* = 0.0631 and females, *P* = 0.0218; hippocampus: males, *P* = 0.0331 and females, *P* = 0.0052) (**Figure 1**). The MAO-B protein was selectively increased in cortex (*P* = 0.0254) and in hippocampus (*P* = 0.0031), and aligned primarily with changes in female samples (**Figure 2**). Representative Western blots for MAO-A and MAO-B in male as well as female cortical and hippocampal samples are included in **Figure 2**.

### MAO-A and MAO-B Activities Based on Diagnosis and *APOE* ε4 Status

We recently reported on the frequency of ε4 carriers across cases in our sample set, which includes ε3/ε3 and ε4/ε4 homozygotes as well as ε2/ε3, ε2/ε4, and ε3/ε4 heterozygotes (Nyarko et al., 2018). A limitation of our sample set is that it does not allow for stratification based on individual APOE allele heterozygosity and homozygosity. Thus, *APOE* ε4 status was used as a dichotomous nominal variable, i.e., a donor was identified as either a carrier (having at least one ε4 allele) or a non-carrier.

**FIGURE 3:**
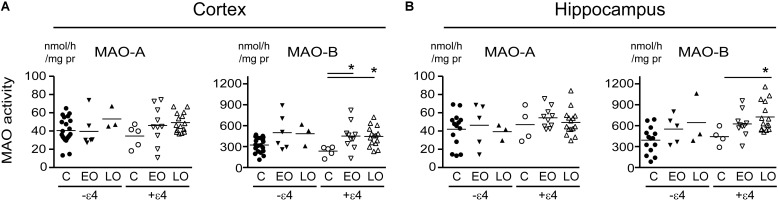
MAO activites in human brain: data stratified by diagnosis-by-*APOE* ε4 status. MAO-A and MAO-B activites were measured in **(A)** cortical and **(B)** the hippocampal samples. Donors with a diagnosis of either Early-Onset AD (EO) or Late-Onset AD (LO), or controls (C) were separated based on their *APOE* ε4 status, i.e., carrier (+ε4) or not (–ε4) of at least one allele. ^∗^*P* < 0.05 between indicated groups.

Any change in MAO-A activity that we observed in our cortical samples (**Figure 1** and noted above) was lost when the data were stratified by diagnosis-by-*APOE* ε4 status (**Figure 3**). In contrast, MAO-B activities were increased in cortex (*P* = 0.0165) and in hippocampus (*P* = 0.0412) in AD donors who carried the ε4 allele. We did observe a trend toward a significant interaction between diagnosis and ε4 status on MAO-B activity in non-carriers in cortex (*P* = 0.1008), but not in hippocampus (*P* = 0.3301).

### MAO-A and MAO-B Activities Based on *APOE* ε4 Status Alone

**FIGURE 4:**
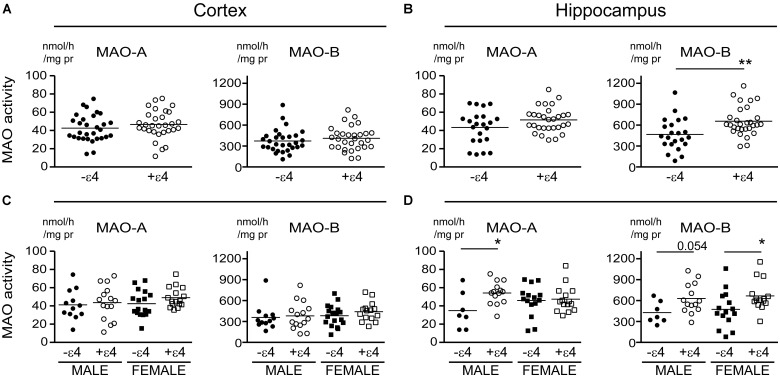
MAO activites in human brain: data stratified by *APOE* ε4 status alone. MAO-A and MAO-B activites were measured in **(A)** the cortex and **(B)** the hippocampus of donors that were carriers (+ε4) or not (–ε4) of an *APOE* ε4 allele. **(C,D)** The same samples were re-analyzed based on the donor’s sex. ^∗^*P* < 0.05; ^∗∗^*P* < 0.01 between indicated groups.

**FIGURE 5:**
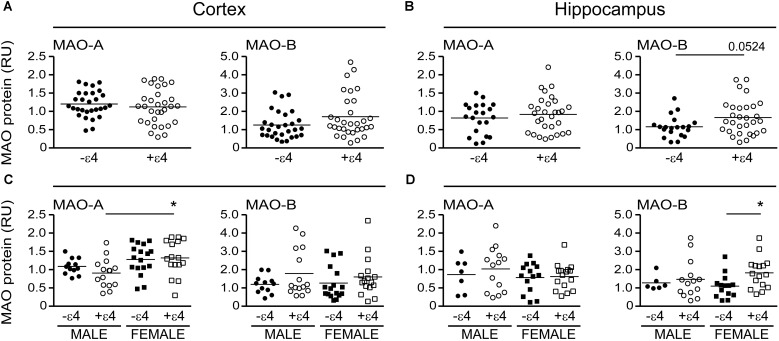
MAO proteins in human brain: data stratified by *APOE* ε4 status alone. MAO-A and MAO-B proteins were quantified by densitometry in **(A)** cortical and **(B)** hippocampal lysates from donors that were carriers (+ε4) or not (–ε4) of an *APOE* ε4 allele. **(C,D)** The same protein densitometries were analyzed based on the donor’s sex.^∗^*P* < 0.05 between indicated groups.

Our recent report suggested that *APOE* ε4 might be exerting effects independently of any diagnosis of AD (Nyarko et al., 2018). Based on that observation, we re-evaluated our data based strictly on the donor’s *APOE* ε4 status (i.e., excluding ‘diagnosis’ as a variable in our analyses). This revealed an increase in MAO-B that was limited to hippocampal samples from carriers of the ε4 allele (*P* = 0.0033), with contributions from males (*P* = 0.0524) as well as females (*P* = 0.0262) (**Figure 4**). Interestingly, we also observed a significant increase in hippocampal MAO-A activity in male carriers of the ε4 allele (*P* = 0.0292). The regional changes in MAO-B activities in carriers of the ε4 allele corresponded to changes in MAO-B protein in the hippocampus (*P* = 0.0524), but not in cortex (*P* = 0.1148) (**Figure 5**).

### MAO-A and MAO-B Activities Based Strictly on the Donor’s Sex

**FIGURE 6:**
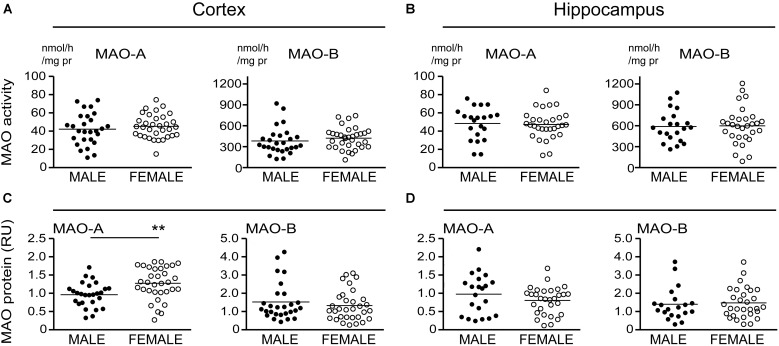
MAO activites in human brain: data stratified by the donor’s sex. **(A)** Cortical and **(B)** hippocampal MAO-A and MAO-B activites were compared based strictly on the donor’s sex. **(C,D)** The corresponding protein densitometries were also analyzed based on the donor’s sex. ^∗∗^*P* < 0.01 between indicated groups.

There is evidence that platelet MAO activity (i.e., MAO-B) increases in women as they age (Robinson et al., 1971; Veral et al., 1997). As such, we examined the regional MAO activities based strictly on the donor’s sex. Comparing sample means between males and females did not reveal any significant difference in MAO-A or MAO-B activities or MAO proteins, except for a significantly higher level of MAO-A protein in female cortical samples (*P* = 0.0019) (**Figure 6**).

Up to this point, our analyses were based on comparing samples means. We did observe parallel increases in MAO-A and MAO-B activity in some of our data sets. For example, we observed increases in both MAO-A and MAO-B activities in the AD cortex (**Figure 1**) and increases in both MAO-A and MAO-B activities in the hippocampus of ε4-positive males (**Figure 4**). We wondered whether any of these changes might be correlated, and whether any potential correlation was influenced by the stratification model used. The summaries of our correlational analyses are provided in Supplementary Tables 2–7. We provide the significant highlights of these analyses below.

### Cortical MAO-A and MAO-B Activities Are Highly Correlated, but This Is Not True for the Hippocampus

**FIGURE 7:**
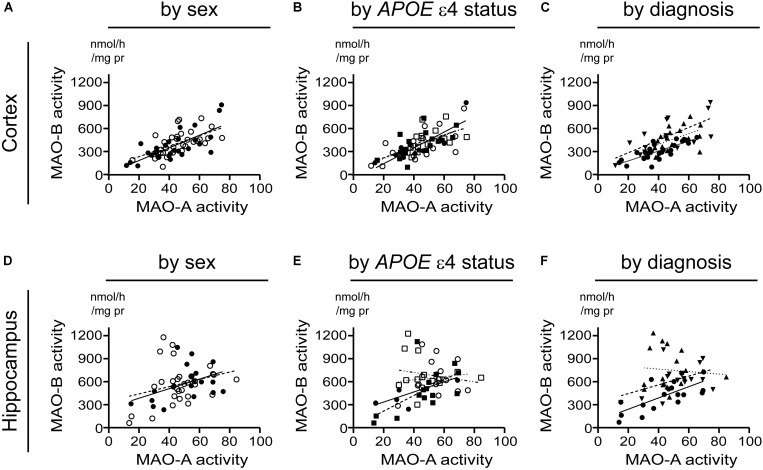
A putative correlation between MAO-A and MAO-B activities in cortex, but not hippocampus, is spared from influence by risk factors for AD. Data are presented as scatter plots and analyses are shown as regression lines (using Pearson’s coefficient). The strength of any potential association between MAO-A and MAO-B activites in our sample set was tested using the three stratifications used for comparing sample means in the previous figures. Cortical data were stratified by **(A)** sex alone (male: 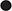, solid line; female: 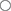, dashed line); **(B)** by *APOE* ε4 status and sex (male/-ε4: 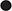, solid line; male/+ε4: 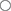, dotted line; female/-ε4: 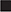, dashed line; female/+ε4: 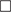, dotdashed line); and **(C)** by diagnosis (control: 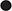, solid line; early-onset AD: 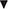, dashed line; late-onset AD: 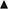 dotted line). The corresponding analyses based on hippocampal data (symbols and line types as for cortical data) are presented by **(D)** sex alone, **(E)** *APOE* ε4-by-sex, and **(F)** diagnosis.

Beginning with the simplest of models, i.e., based strictly on the donor’s sex, we found that cortical MAO-A and MAO-B activities were significantly correlated in males (*P* < 0.0001, *r* = 0.730) and females (*P* = 0.0004, *r* = 0.581). While hippocampal MAO-A and MAO-B activities were significantly correlated in males (*P* = 0.0252, *r* = 0.487), they were not correlated in females (*P* = 0.1352) (**Figures 7A,D**). Focussing on *APOE* ε4 status revealed that MAO-A and MAO-B activities in the cortical samples were correlated in non-carriers of the ε4 allele (males, *P* = 0.0005, *r* = 0.846; females, *P* = 0.0082, 0.622) as well as in carriers (males, *P* = 0.0058, *r* = 0.654; females, *P* = 0.0848, *r* = 0.460). In contrast, hippocampal MAO-A and MAO-B activities were correlated in non-carriers of the ε4 allele (males, *P* = 0.0403, *r* = 0.776; females, *P* = 0.0154, *r* = 0.612), but not in those donors that did carry the allele (males, *P* = 0.7079; females, *P* = 0.4963) (**Figures 7B,E**). Finally, stratifying for ‘diagnosis’ (i.e., control *versus* EOAD or LOAD) revealed that MAO-A and MAO-B activities were correlated in cortical control (*P* < 0.0001, *r* = 0.784), EOAD (*P* = 0.0027, *r* = 0.696), and LOAD (*P* = 0.0419, *r* = 0.484) samples. Once again, we observed a different pattern in hippocampal MAO-A and MAO-B activities; indeed, they were correlated in control samples (*P* = 0.0012, *r* = 0.702), less so in EOAD samples (*P* = 0.0827, *r* = 0.462), and not at all in LOAD samples (*P* = 0.7255) (**Figures 7C,F**).

### MAO-A and MAO-B Proteins Are Generally Not Correlated, Regardless of the Sex, *APOE* ε4 Status, or Diagnosis of the Donor

**FIGURE 8:**
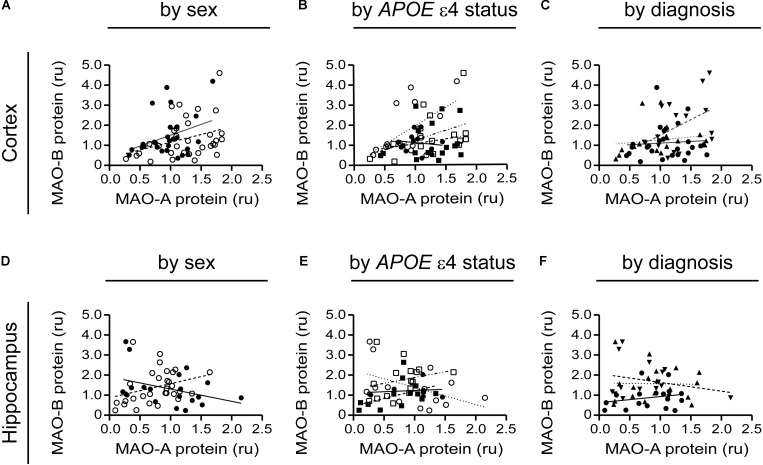
MAO-A and MAO-B proteins are generally not correlated in either the cortex or hippocampus. Cortical data were stratified by **(A)** sex alone (male: 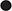, solid line; female: 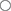, dashed line); **(B)** by *APOE* ε4 status and sex (male/-ε4: 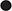, solid line; male/+ε4: 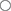, dotted line; female/-ε4: 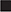, dashed line; female/+ε4: 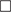, dotdashed line); and **(C)** by diagnosis (control: 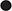, solid line; early-onset AD: 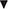, dashed line; late-onset AD: 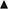 dotted line). The corresponding analyses revealed a similar lack of correlation in hippocampal data (symbols and line types as for cortical data) whether analyzed by **(D)** sex alone, **(E)** *APOE* ε4-by-sex, or **(F)** diagnosis.

A tendency for correlation in the cortex (males: *P* = 0.0822; females: *P* = 0.0571) (if the data are stratifi**Figure 8A**) and in cortical samples from male non-carriers of the ε4 allele (*P* = 0.0299) (**Figure 8B**) were observed. Aside from these few exceptions, MAO-A and MAO-B protein expression levels did not correlate, regardless of the stratification model used or the region being tested (**Figures 8C–F**).

### *MAO-A* and *MAO-B* mRNA Transcript Levels Are Correlated in the Cortex, but Not in the Hippocampus

**FIGURE 9:**
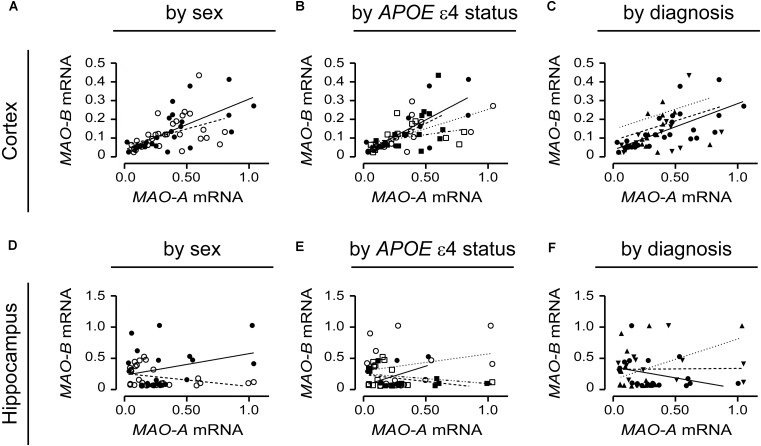
*MAO-A* and *MAO-B* mRNA transcript levels are correlated in the cortex. Cortical data were stratified by **(A)** sex alone (male: 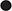, solid line; female: 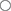, dashed line); **(B)** by *APOE* ε4 status and sex (male/-ε4: 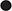, solid line; male/+ε4: 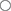, dotted line; female/-ε4: 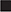, dashed line; female/+ε4: 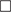, dotdashed line); and **(C)** by diagnosis (control: 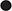, solid line; early-onset AD: 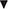, dashed line; late-onset AD: 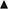 dotted line). The corresponding analyses of hippocampal data (symbols and line types as for cortical data) presented by **(D)** sex alone, **(E)** *APOE* ε4-by-sex, and **(F)** diagnosis revealed a different pattern.

*MAO-A* and *MAO-B* mRNA transcript levels were compared in the simplest models, i.e., based strictly on the donor’s sex. *MAO-A* and *MAO-B* mRNAs were significantly correlated in males (*P* = 0.0001, *r* = 0.687) and females (*P* = 0.0083, *r* = 0.466) in the cortex (**Figure 9A**). However, there was no correlation in the corresponding male hippocampal samples (*P* = 0.1992; **Figure 9D**), while a tendency for a correlation was observed for female hippocampal samples (*P* = 0.0650, *r* = 0.353) (**Figure 9B**). Focussing on *APOE* ε4 status revealed that cortical *MAO-A* and *MAO-B* mRNAs were correlated in non-carriers of the ε4 allele (males, *P* = 0.0045, *r* = 0.754; females, *P* = 0.0198, *r* = 0.575) and in male (*P* = 0.0126, *r* = 0.646), but not female (*P* = 0.1636), carriers of the allele (**Figure 9B**). In contrast, *MAO-A* and *MAO-B* mRNAs were not correlated in the corresponding hippocampal samples when stratified for *APOE* ε4 status (**Figure 9E**). Finally, stratifying for ‘diagnosis’ revealed that *MAO-A* and *MAO-B* mRNAs were correlated in cortical control samples (*P* < 0.0001, *r* = 0.691), but not in EOAD (*P* = 0.3924) and LOAD (*P* = 0.6147) samples (**Figure 9C**). Hippocampal *MAO-A* and *MAO-B* mRNAs were not correlated in any diagnosis using this stratification model (**Figure 9F**).

### MAO Activities and Proteins Are Generally Not Correlated

**FIGURE 10:**
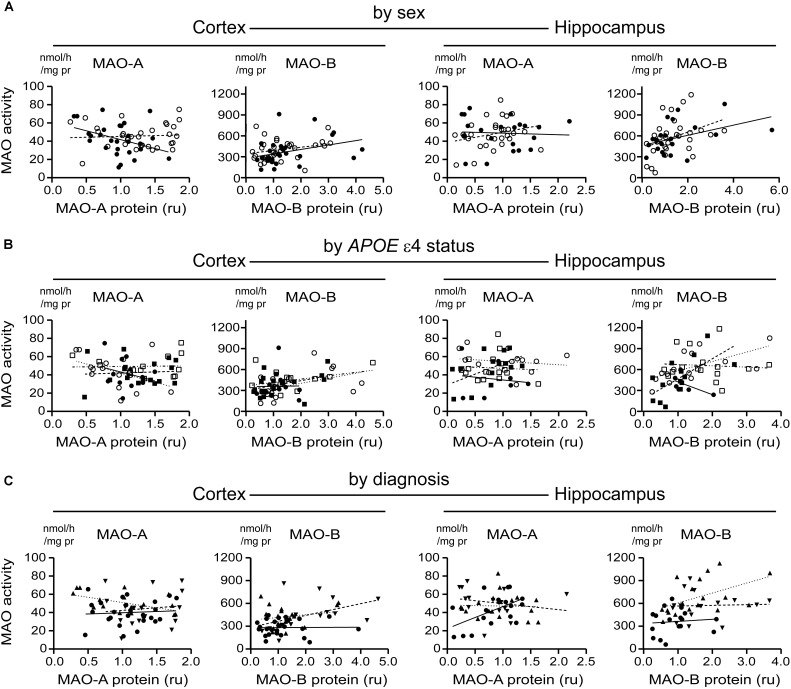
MAO-A and MAO-B activities do not correlate with their corresponding proteins in either the cortex or the hippocampus. Cortical and hippocampal data were stratified by **(A)** sex alone (male: 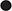, solid line; female: 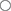, dashed line); **(B)** by *APOE* ε4 status and sex (male/-ε4: 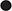, solid line; male/+ε4: 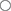, dotted line; female/-e4: 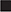, dashed line; female/+ε4: 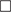, dotdashed line); and **(C)** by diagnosis (control: 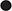, solid line; early-onset AD: 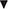, dashed line; late-onset AD: 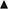 dotted line). In general, there was no correlation between activities and respective proteins.

We did observe a correlation between hippocampal MAO-B activity and protein expression in males (*P* = 0.0579) and females (*P* = 0.0455) and a negative correlation between MAO-A activity and protein expression in male cortical samples (*P* = 0.0558) (**Figure 10A**). When stratifying for *APOE* ε4 status, we did observe a strong correlation between MAO-B activity and protein expression in hippocampal samples from female non-carriers of the ε4 allele (*P* = 0.0094) (**Figure 10B**). Finally, there was a correlation between MAO-A activity and protein expression in control hippocampal samples (*P* = 0.0214) and a negative correlation between MAO-A activity and protein expression in cortical LOAD samples (*P* = 0.0244) (**Figure 10C**). Aside from these exceptions, all other regression analyses did not reveal any significant patterns. We were unable to demonstrate any correlation between MAO-A and MAO-B protein expression and respective mRNA transcript levels (*in the interest of space, these data are not shown*).

### Overexpression of Human APOE4 Increases MAO-A and MAO-B Activities in C6 and HT-22 Cultures

**FIGURE 11:**
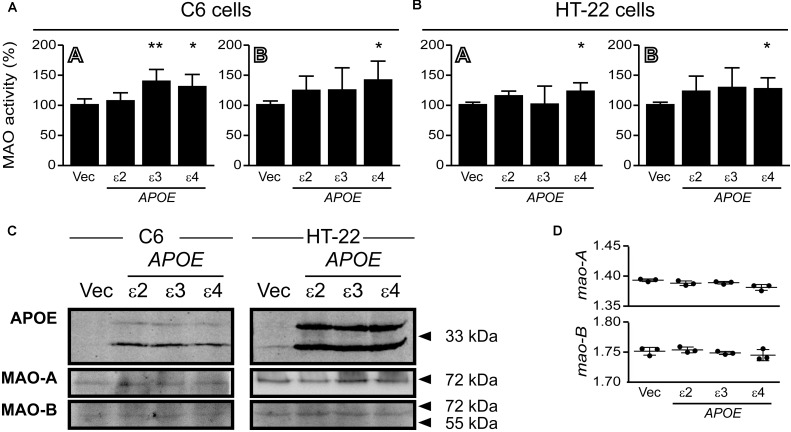
Overexpressed APOE4 influences MAO activity in cell cultures. C6 (glial) and HT-22 (neuronal) cells were transfected with cDNA coding for the human *APOE* ε2, ε3, or ε4 allele. Overexpressed APOE4 induced MAO-A and MAO-B activity in **(A)** C6 cultures and in **(B)** HT-22 cultures. The APOE3 variant induced MAO-A activity specifically in C6 cells. **(C)** Representative blots of the overexpressed APOE variants and MAO-A and MAO-B. **(D)** The APOE variants did not elicit any change in C6 *mao-A* or *mao-B* mRNA transcript levels. ^∗^*P* < 0.05; ^∗∗^*P* < 0.01 relative to the Vector (Vec) transfected control group.

C6 glial cells and hippocampal HT-22 cells were transfected with cDNA coding for human APOE2, APOE3, and APOE4 for 24 h. There was a significant increase in MAO-A activity in C6 cells overexpressing APOE3, while APOE4 expression led to increases in both MAO-A and MAO-B activities in both cell lines (**Figure 11**). The overexpressed APOE variants did not affect endogenous MAO protein or mRNA expression in either cell line (**Figure 11**).

### Levels of MAO-Mediated Metabolites and Substrates

Aside from a tendency for an increase in hippocampal HVA levels in females with a diagnosis of EOAD/LOAD (vs. control females) (*P* = 0.0824), significantly more HVA in the cortex of female (vs. male) carriers of the ε4 allele (*P* = 0.0564) and significantly more 5-HIAA in the hippocampus of female (vs. male) carriers of the ε4 allele (*P* = 0.0194). There were generally no differences in the levels of either 5-HT or dopamine, or their respective metabolites 5-HIAA, or HVA (*data not shown*).

## Discussion

One of the strengths of this study was our ability to compare data generated from cortical and corresponding hippocampal samples from a given donor; this allowed us to assess whether region-dependent differences existed in any of the patterns we observed for a given MAO parameter.

### MAO Indices and the Risk of AD

The stratification based on the donor’s diagnosis (i.e., control vs. AD) revealed the anticipated regional changes in MAO activities. Increased MAO-A activity in the AD brain has been associated with prodromal and co-morbid neuropsychiatric symptoms (Mousseau and Baker, 2012) and with neurodegeneration (Naoi et al., 2012). Similarly, region-specific changes in MAO-B activity in the AD brain have been observed (Adolfsson et al., 1980; Oreland and Gottfries, 1986) and MAO-B-positive astrocytes have been localized to the amyloid plaque in human brain (Saura et al., 1994) as well as in the brain of a mouse model of AD (Kim et al., 2016). Some of these changes are region-dependent, which could be reflecting different roles attributed to MAO-A in a given disease. For example, changes in cortical MAO-A binding in humans have been implicated in depression (Meyer et al., 2006), whereas changes in hippocampal MAO-A (based on animal models) have been associated with changes in cognition (Steckler et al., 2001) and even motor activity (Morishima et al., 2006), which is more often associated with the dopaminergic system. In fact, cerebrospinal fluid levels of the MAO-mediated dopamine metabolite, i.e., HVA, have been used to propose different subtypes of AD (Sjogren et al., 2002). Our current data suggest that HVA changes might be more evident in the female (vs. male) hippocampus (whether stratified by diagnosis or by *APOE* ε4 status). MAO-B appears to contribute to oxidative stress and motor deficits associated with AD, parkinsonism, and aging (Fowler et al., 1980; Riederer and Jellinger, 1982; Oreland and Gottfries, 1986; Danielczyk et al., 1988). In addition, MAO-B inhibitors might reduce the generation of the Aβ peptide from the APP precursor (Huang et al., 2012) by increasing non-amyloidogenic APP processing *via* α-secretase (Yang et al., 2009). MAO function, however, is complex (Mousseau and Baker, 2012) and our inability to correlate MAO-A (or -B) activity with MAO protein expression (or with mRNA expression) in our brain sample set, while perplexing, is not unique. Indeed, mean platelet MAO-B activity has been shown to correlate with MAO-B mRNA and protein expression (Zellner et al., 2012) and to increase in female AD patients (Robinson et al., 1971; Veral et al., 1997), while MAO-A or MAO-B activities tend to correlate with the respective proteins or binding densities (reviewed in (Tong et al., 2013)). Yet, in contrast to these observations, *MAO-A* genotypes that influence transcriptional activity (Fowler et al., 2007) and [^11^C]-harmane/MAO-A autoradiography in autopsied human brain (Tong et al., 2013) do not reflect levels of MAO-A activity. We now know that some of these discrepancies might be reflecting possible post-translational modifications based on, for example, phosphorylation (Cao et al., 2009b), calcium-binding (Kosenko et al., 2003; Cao et al., 2007, 2009a), aberrant subcellular localization (Gujrati et al., 1996), and/or a direct interaction with a binding partner, including the presenilin protein (Pennington et al., 2011; Wei et al., 2012; Schedin-Weiss et al., 2017) that is central to Aβ/AD-related changes in γ-secretase activity (Roychaudhuri et al., 2009).

While the stratification based on diagnosis corroborated previous reports, we were far more intrigued by what other stratifications –based on putative risk factors for AD– revealed about MAO-A and MAO-B function. Given that the *APOE* ε4 allele has been linked to AD in a Brazilian cohort *via* co-segregation with a *MAO-A* polymorphism and an allelic variant of the serotonin transporter (Nishimura et al., 2005) and that a multiplex protein biochip for screening for AD –based on the ε4 allele and MAO-B– has been proposed (Veitinger et al., 2014), there is surprisingly very little published on the interaction between *APOE* risk alleles and MAO.

The most robust findings that we observed were the significant increases in MAO-B activities in the cortex and hippocampus of carriers of the *APOE* ε4 allele. Yet, tendencies for an increase were also observed in non-carriers of the allele (although the small sample size in these groups might have mitigated statistical significance). When ‘diagnosis’ was excluded as a nominal variable, we observed a male-specific increase in hippocampal MAO-A activity and an increase in hippocampal MAO-B activity in both males and females. This is surprising given the female-specific risk associated with the ε4 allele in pathologies such as AD (Poirier et al., 1993) and depression (Delano-Wood et al., 2008). We are unsure what this observation implies, but perhaps it is revealing a more subtle role for the APOE4 protein in males as already shown for sexual dimorphism in neurogenesis (Rijpma et al., 2013), intelligence (Mondadori et al., 2007), or enhanced damage due to stroke (DeCarli et al., 1999). In our C6 and HT-22 cell cultures, overexpression of the human APOE4 protein led to increases in mao-A and mao-B activities (without any concurrent change in MAO protein), which suggest that APOE4 might exert a post-translational influence on MAO function and/or associated pathologies or potentially in very specific populations of AD patients, for example, the Brazilian cohort (Nishimura et al., 2005). This certainly deserves further attention.

### A Putative Co-regulation of MAO-A and MAO-B Is Vulnerable to AD Risk Factors in the Hippocampus, but Not in the Cortex

The outcomes discussed in the previous section, based on comparing sample means, generally confirmed what has been reported in the literature. We have recently reported that variability in regional Aβ levels in carriers of the *APOE* ε4 allele correlated with the variability in the expression of catalytic enzymes implicated in the generation of the Aβ peptide (Nyarko et al., 2018). We re-examined our data so as to determine whether any of the parallel increases observed in MAO-A and MAO-B parameters quantified up to this point might be indicating a more subtle relation and, if so, whether any potential correlation was influenced by risk factors or AD itself. While there have been plenty of studies looking at the correlation between MAO activity and physiological correlates such as age or with behavioral correlates such as risk-seeking, drug dependence and/or criminality (Mousseau and Baker, 2012), or specifically between platelet MAO activity and protein expression (Zellner et al., 2012), we were surprised, upon review of the literature, that there have only been a few reports suggesting that the function of the two MAO isoforms might be correlated. An earlier study had found a correlation between MAO-A and MAO-B activity in surgical resection samples (Young et al., 1986), while a more recent study found that MAO-A and MAO-B activities were correlated in the prefrontal cortex of controls and marginally less-so in AD samples (Kennedy et al., 2003).

We began with the ‘simplest’ stratification, i.e., focussing on biological sex, and progressed toward the more complex stratifications, e.g., including *APOE* ε4 status or ‘diagnosis.’ We observed that the activities of MAO-A and MAO-B in the cortex were highly correlated in males and females, and that this correlation generally held even when the data were stratified for sex-by-*APOE* ε4 status, or for a diagnosis of EOAD or LOAD. Interestingly, any correlation in the hippocampal data was limited to males (if stratifying for sex alone), to individuals who did not carry an ε4 allele (regardless of sex), or to individuals who did not have a diagnosis of EOAD or LOAD. In other words the co-regulation of MAO-A and MAO-B activities was maintained in the cortex regardless of risk or diagnosis of AD, but was disrupted in the hippocampus by these same risk factors for AD, i.e., sex (female) or *APOE* ε4 allele, or in donors with a confirmed diagnosis of AD. Parenthetically, it would be interesting to determine how much of the blockade of MAO-A activity that is observed following chronic MAO-B inhibition is due to any disruption of this co-regulatory mechanism rather than to changes in substrate specificity or an off-target effect of the drug, as often surmised (Inaba-Hasegawa et al., 2012; Bartl et al., 2014).

The fact that the MAO-A and MAO-B proteins did not appear to correlate at all (regardless of region or stratification) is perhaps not surprising given that region-dependent factors such as post-translational modifications, availability of Ca^2+^-binding proteins, and physical interactions with other cellular proteins all could be regulating protein activity and/or availability at any given time (discussed above). The fact that *MAO-A* and *MAO-B* mRNA were highly co-regulated in cortical, but not hippocampal, samples is certainly more intriguing. We do not have an explanation for this phenomenon at the moment, but could speculate on region-dependent influences, perhaps involving sex-dependent differences in hormonal signaling pathways (Barth et al., 2015; Bethea et al., 2015), epigenetics (direct or indirect) *via* clock genes, microRNA (e.g., miR-142) or differential methylation of the *MAO-A* and *MAO-B* genes (Hampp et al., 2008; Shumay et al., 2012; Chaudhuri et al., 2013; Tiili et al., 2017), or even the possibility of transcriptional *trans*-regulation. The latter is not an unreasonable scenario given that we observed that the overexpression of human MAO-A is able to reduce *mao-B* –but not *mao-A*– mRNA transcript levels in C6 cells (preliminary observations; data not shown). The possibility of a co-regulation of *MAO-A* and *MAO-B* transcription by any of these factors, or combinations thereof, will add to our understanding of the promoter activities of these two genes (Bach et al., 1988; Zhu et al., 1992).

Monoamines play a significant role in cognition, which likely reflects their anatomical association with those brain areas regulating memory and learning (King et al., 2008). Aberrant monoaminergic (i.e., serotoninergic) signaling usually accompanies cholinergic deficits (Grailhe et al., 2009), which supports early-stage changes in monoaminergic tone, compounded by cholinergic deficits, as contributing factors to the cognitive decline in AD (Robinson, 1983; Richter-Levin and Segal, 1993). Selective changes in monoaminergic function could be a major contributor to non-cognitive symptoms, including depression (Ritchie and Lovestone, 2002), which could account for prodromal depression, as mentioned above. Some of these earlier stages of AD are often associated with non-cognitive, neuropsychiatric symptoms including depression, irritability, aggressive outbursts, and delusions (Ritchie and Lovestone, 2002). It is well known that these disorders reflect region-specific noradrenergic and serotoninergic insults. Thus, MAO-A and -B dysfunction as well as a monoaminergic insult could be contributing to a spectrum of symptoms and pathology spanning earlier –and sustained– AD progression.

We acknowledge that correlation does not mean causation; yet, given that MAO-B inhibitors have been shown to reduce the amyloidogenic processing of the APP molecule (Yang et al., 2009; Huang et al., 2012), this would imply that an increase in MAO-B function would promote amyloidogenic processing of APP. This could explain the well-documented increase in Aβ levels and plaque burden in the AD hippocampus, where an increase in MAO-B has been observed. Unfortunately, the role for MAO in the context of AD progression is not likely to be as straightforward as anticipated given that Roche recently terminated a clinical trial centered on sembragiline, a highly selective MAO-B inhibitor, because of a lack of amelioration of the Alzheimer’s Disease Assessment Scale-Cognitive Behavior Subscale^2^.

Any increase in MAO function would lead to the generation of hydrogen peroxide as a by-product of the deamination reaction, and the ensuing oxidative stress and potential for cell death –invariably involving the mitochondria– would be exacerbated when antioxidant systems are compromised, such as during aging (Zhu et al., 2006) and particularly in AD (Crack et al., 2006).

## Conclusion

Our study, based on post-mortem brain tissue, highlights a region-dependent MAO (co)regulation. While we could not fully characterize the role of this co-regulation in the male and female brain, the findings do provide insight into the role of MAOs –and the monoaminergic neurotransmitters that they regulate– and support sex– and genetic-specific responses to risk of AD and the pathology associated with disease progression.

Our observations confirm some of what is already known, but certainly expand on the reported literature. For instance, the increases in MAO-B activity that appear to align much more strongly with carriers of the *APOE* ε4 allele is novel and given the implied role of MAO-B in neurodegeneration and the gender-risk of AD associated with the *APOE* ε4 allele, it is not unreasonable to infer that their contributions to AD reflect an overlapping mechanism. What our data also provide is a side-by-side comparison of two brain regions, namely the cortex and the hippocampus, from the same donor. Again, the fact that many of the changes in MAO-B (and MAO-A) are occurring in the hippocampus –a region particularly vulnerable during the course of AD– is supportive of a contribution of these enzymes to disease progression. What our data are also revealing is that it is important to test for activity, protein expression, and mRNA expression before one can truly determine a role for MAOs in any pathological (or physiological) state. As importantly, or perhaps more so, our observations also highlight the limitation(s) of only examining one or the other MAO isoform, and/or using sample means to compare between test groups; indeed, something as obvious as a co-regulation of the two isoforms has been inadvertently overlooked. The fact that this co-regulation of MAO-A and MAO-B is sex-dependent and apparently invariant in the cortex, but so vulnerable to risk factors for AD in the hippocampus (observed using samples from the same donors), strongly suggests a contribution to AD progression. Whether this co-regulation contributes to other neuropathologies such as Parkinson’s disease, depression, autism –or any other disorder associated with monoaminergic defects– remains to be determined, but could certainly explain some of the ambiguity in neurochemical underpinnings and treatment responses associated with these various diseases in the clinic. This new-found knowledge relating to two enzymes that are also important to oxidative stress and mitochondria-associated processes in the central nervous system as well as in the periphery will provide fundamental and critically important insight into their implied roles in the research and clinical contexts.

## Author Contributions

MQ, JN, PP, RH, and PK: data collation. MQ, JN, GB, and DM: experimental design, and manuscript preparation and editing (all authors).

## Conflict of Interest Statement

The authors declare that the research was conducted in the absence of any commercial or financial relationships that could be construed as a potential conflict of interest.

## Footnotes

1 http://rsb.info.nih.gov/ij/

2 http://www.alzforum.org/therapeutics/sembragiline
