# Supplementary material for: Alzheimer Disease and Selected Risk Factors Disrupt a Co-regulation of Monoamine Oxidase-A/B in the Hippocampus, but Not in the Cortex
Source: Front Neurosci. 2018 Jun 26;12:419. doi: 10.3389/fnins.2018.00419 (PMC6029266; doi:10.3389/fnins.2018.00419)
Supplement: Supplementary file 1 [file Table_1.PDF]

Table of Contents

Table 1: Donor Statistics

<u>Legend for **Tables 2** to **7**:</u>

MAO	Monoamine oxidase
APOE	Apolipoprotein E
-ε4	Non-carriers of the epsilon4 allele of Apolipoprotein E
+ε4	Carriers of the epsilon4 allele of Apolipoprotein E
CTL	Control
EOAD	Early-onset Alzheimer disease
LOAD	Late-onset Alzheimer disease
Pooled	Male + Female donors

Table 2: Linear regression (based on Pearson's correlation coefficient) between MAO-A/MAO-B variables: ***by sex***

(i) Linear regression between MAO-A ***activity*** and MAO-B ***activity***

(ii) Linear regression between MAO-A ***protein*** and MAO-B ***protein***

(iii) Linear regression between MAO-A ***mRNA*** and MAO-B ***mRNA***

Table 3: Linear regression (based on Pearson's correlation coefficient) between the activity of MAO isoforms and their respective proteins: ***by sex***

(i) Linear regression between MAO-A ***activity*** and MAO-A ***protein***

(ii) Linear regression between MAO-B ***activity*** and MAO-B ***protein***

Table 4: Linear regression (based on Pearson's correlation coefficient) between MAO-A/MAO-B variables: ***by APOE ε4 status and sex***

(i) Linear regression between MAO-A ***activity*** and MAO-B ***activity***

(ii) Linear regression between MAO-A ***protein*** and MAO-B ***protein***

(iii) Linear regression between MAO-A ***mRNA*** and MAO-B ***mRNA***

Table 5: Linear regression (based on Pearson's correlation coefficient) between the activity of MAO isoforms and their respective proteins: ***by APOE ε4 status and sex***

(i) Linear regression between MAO-A ***activity*** and MAO-A ***protein***

(ii) Linear regression between MAO-B ***activity*** and MAO-B ***protein***

Table 6: Linear regression (based on Pearson's correlation coefficient) between MAO-A/MAO-B variables: ***by diagnosis***

(i) Linear regression between MAO-A ***activity*** and MAO-B ***activity***

(ii) Linear regression between MAO-A ***protein*** and MAO-B ***protein***

(iii) Linear regression between MAO-A ***mRNA*** and MAO-B ***mRNA***

Table 7: Linear regression (based on Pearson's correlation coefficient) between the activity of MAO isoforms and their respective proteins: ***by diagnosis***

(i) Linear regression between MAO-A ***activity*** and MAO-A ***protein***

(ii) Linear regression between MAO-B ***activity*** and MAO-B ***protein***

Table 1: Donor Statistics

		Control (26)	EOAD (16)	LOAD (18)
Sex	M/F	12/14	7/9	8/10
Age, yr	M+F	70.73 ± 12.49	58.13 ± 7.80*	83.17 ± 5.77**
	M	70.67 ± 9.85	63.14 ± 5.61	82.88 ± 5.38*
	F	70.79 ± 14.76	54.22 ± 7.16*	83.40 ± 6.35
PMI, h	M+F	19.26 ± 10.98	21.44 ± 10.42	21.41 ± 9.06
	M	16.66 ± 8.50	25.21 ± 10.58	22.66 ± 8.54
	F	21.67 ± 12.73	18.51 ± 9.87	20.40 ± 7.98
Brain weight, g	M+F	1232 ± 136.5	1016 ± 200.1**	1036 ± 121.9***
	M	1275 ± 155.1	1157 ± 139.2	1126 ± 98.9*
	F	1191 ± 108.6	879.4 ± 143.4***	992.0 ± 94.9*

*: P < 0.05

**: P < 0.01

***: P < 0.001

P values are reported versus control donors.

EOAD	Early-onset Alzheimer disease
LOAD	Late-onset Alzheimer disease
PMI	Post-mortem interval

Table 2: Linear regression (based on Pearson's correlation coefficient) between MAO-A/MAO-B variables: ***by sex***

(i) Linear regression between MAO-A *activity* and MAO-B *activity*

Cortex

	**Male**	**Female**
**Slope**	7.849 ± 1.469	6.520 ± 1.641
**Goodness of Fit**		
R value	0.7303	0.5809
P value	< **0.0001**	**0.0004**
Deviation from zero?	Significant	Significant
**Total number of values**	27	33

Hippocampus

	**Male**	**Female**
**Slope**	5.958 ± 2.452	4.522 ± 2.939
**Goodness of Fit**		
R value	0.4869	0.2792
P value	**0.0252**	0.1351
Deviation from zero?	Significant	Not Significant
**Total number of values**	21	30

(ii) Linear regression between MAO-A *protein* and MAO-B *protein*

Cortex

	**Male**	**Female**
**Slope**	1.060 ± 0.5853	0.7629 ± 0.3860
**Goodness of Fit**		
R value	0.3404	0.3345
P value	0.0822	0.0571
Deviation from zero?	Not Significant	Not Significant
**Total number of values**	27	33

Hippocampus

	**Male**	**Female**
**Slope**	-0.6025 ± 0.3475	0.6945 ± 0.4000
**Goodness of Fit**		
R value	0.3783	0.3118
P value	0.1001	0.0935
Deviation from zero?	Not Significant	Not Significant
**Total number of values**	20	30

(iii) Linear regression between MAO-A *mRNA* and MAO-B *mRNA*

Cortex

	**Male**	**Female**
**Slope**	0.2890 ± 0.06235	0.1936 ± 0.06833
**Goodness of Fit**		
R value	0.6872	0.4656
P value	**0.0001**	**0.0083**
Deviation from zero?	Significant	Significant
**Total number of values**	26	31

Hippocampus

	**Male**	**Female**
**Slope**	0.3681 ± 0.2767	-0.2198 ± 0.1141
**Goodness of Fit**		
R value	0.2919	0.3534
P value	0.1992	0.0650
Deviation from zero?	Not Significant	Not Significant
**Total number of values**	21	28

Table 3: Linear regression (based on Pearson's correlation coefficient) between the activity of MAO isoforms and their respective proteins: ***by sex***

(i) Linear regression between MAO-A *activity* and MAO-A *protein*

Cortex

	**Male**	**Female**
**Slope**	-19.40 ± 9.671	1.777 ± 5.295
**Goodness of Fit**		
R value	0.3723	0.0601
P value	0.0558	0.7395
Deviation from zero?	Not Significant	Not Significant
**Total number of values**	27	33

Hippocampus

	**Male**	**Female**
**Slope**	-1.716 ± 7.304	10.96 ± 7.538
**Goodness of Fit**		
R value	0.0538	0.2650
P value	0.8167	0.1570
Deviation from zero?	Not Significant	Not Significant
**Total number of values**	21	30

(ii) Linear regression between MAO-B *activity* and MAO-B *protein*

Cortex

	**Male**	**Female**
**Slope**	60.46 ± 33.90	44.12 ± 29.62
**Goodness of Fit**		
R value	0.3360	0.2624
P value	0.0866	0.1469
Deviation from zero?	Not Significant	Not Significant
**Total number of values**	27	32

Hippocampus

	**Male**	**Female**
**Slope**	69.33 ± 34.35	110.6 ± 52.86
**Goodness of Fit**		
R value	0.4202	0.3678
P value	0.0579	**0.0455**
Deviation from zero?	Not Significant	Significant
**Total number of values**	21	30

Table 4: Linear regression (based on Pearson's correlation coefficient) between MAO-A/MAO-B variables: ***by APOE ε4 status and sex***

(i) Linear regression between MAO-A *activity* and MAO-B *activity*

Cortex

	Male		Female	
	**-ε4**	**+ε4**	**-ε4**	**+ε4**
**Slope**	9.449 ± 1.886	6.862 ± 2.201	6.758 ± 2.124	5.577 ± 2.989
**Goodness of Fit**				
R value	0.8456	0.6541	0.6225	0.4596
P value	**0.0005**	**0.0082**	**0.0058**	0.0848
Deviation from zero?	Significant	Significant	Significant	Not Significant
**Total number of values**	12	15	18	15

Hippocampus

	Male		Female	
	**-ε4**	**+ε4**	**-ε4**	**+ε4**
**Slope**	6.015 ± 2.186	1.912 ± 4.983	9.290 ± 3.331	-2.782 ± 3.975
**Goodness of Fit**				
R value	0.7760	0.1101	0.6118	0.1906
P value	**0.0403**	0.7079	**0.0154**	0.4963
Deviation from zero?	Significant	Not Significant	Significant	Not Significant
**Total number of values**	7	14	15	15

(ii) Linear regression between MAO-A *protein* and MAO-B *protein*

Cortex

	Male		Female	
	**-ε4**	**+ε4**	**-ε4**	**+ε4**
**Slope**	-0.2887 ± 0.7026	1.834 ± 0.7523	0.4690 ± 0.5576	0.9663 ± 0.5526
**Goodness of Fit**				
R value	0.1288	0.5602	0.2122	0.4233
P value	0.6899	**0.0299**	0.4135	0.1023
Deviation from zero?	Not Significant	Significant	Not Significant	Not Significant
**Total number of values**	12	15	17	16

Hippocampus

	Male		Female	
	**-ε4**	**+ε4**	**-ε4**	**+ε4**
**Slope**	0.1200 ± 0.4179	-0.8435 ± 0.4430	0.7413 ± 0.4105	0.5680 ± 0.6122
**Goodness of Fit**				
R value	0.1421	0.4817	0.4623	0.2406
P value	0.7883	0.0812	0.0961	0.3693
Deviation from zero?	Not Significant	Not Significant	Not Significant	Not Significant
**Total number of values**	6	14	14	16

(iii) Linear regression between MAO-A *mRNA* and MAO-B *mRNA*

Cortex

	Male		Female	
	**-ε4**	**+ε4**	**-ε4**	**+ε4**
**Slope**	0.3732 ± 0.1029	0.2177 ± 0.07432	0.3529 ± 0.1342	0.09736 ± 0.06594
**Goodness of Fit**				
R value	0.7538	0.6458	0.5749	0.3789
P value	**0.0046**	**0.0126**	**0.0198**	0.1636
Deviation from zero?	Significant	Significant	Significant	Not Significant
**Total number of values**	12	14	16	15

Hippocampus

	Male		Female	
	**-ε4**	**+ε4**	**-ε4**	**+ε4**
**Slope**	0.6607 ± 0.6012	0.2898 ± 0.3395	-0.2333 ± 0.1348	-0.1683 ± 0.1988
**Goodness of Fit**				
R value	0.4411	0.2392	0.4802	0.2207
P value	0.3218	0.4101	0.1141	0.4114
Deviation from zero?	Not Significant	Not Significant	Not Significant	Not Significant
**Total number of values**	7	14	12	16

Table 5: Linear regression (based on Pearson's correlation coefficient) between the activity of MAO isoforms and their respective proteins: ***by APOE ε4 status and sex***

(i) Linear regression between MAO-A *activity* and MAO-A *protein*

Cortex

	Male		Female	
	**-ε4**	**+ε4**	**-ε4**	**+ε4**
**Slope**	-19.41 ± 22.96	-20.06 ± 11.96	2.298 ± 8.875	0.6908 ± 6.088
**Goodness of Fit**				
R value	0.2582	0.4218	0.0667	0.0303
P value	0.4177	0.1174	0.7992	0.9113
Deviation from zero?	Not Significant	Not Significant	Not Significant	Not Significant
**Total number of values**	12	15	17	16

Hippocampus

	Male		Female	
	**-ε4**	**+ε4**	**-ε4**	**+ε4**
**Slope**	-6.077 ± 19.41	-3.551 ± 5.801	23.11 ± 10.66	-2.395 ± 10.36
**Goodness of Fit**				
R value	0.1387	0.1740	0.5305	0.0617
P value	0.7669	0.5518	0.0510	0.8205
Deviation from zero?	Not Significant	Not Significant	Not Significant	Not Significant
**Total number of values**	7	14	14	16

(ii) Linear regression between MAO-B *activity* and MAO-B *protein*

Cortex

	Male		Female	
	**-ε4**	**+ε4**	**-ε4**	**+ε4**
**Slope**	4.651 ± 118.5	69.18 ± 37.65	66.07 ± 40.66	45.98 ± 30.25
**Goodness of Fit**				
R value	0.0124	0.4541	0.3869	0.3764
P value	0.9695	0.0891	0.1249	0.1508
Deviation from zero?	Not Significant	Not Significant	Not Significant	Not Significant
**Total number of values**	12	15	17	16

Hippocampus

	Male		Female	
	**-ε4**	**+ε4**	**-ε4**	**+ε4**
**Slope**	-174.5 ± 119.7	128.6 ± 45.10	274.4 ± 88.88	-18.00 ± 66.74
**Goodness of Fit**				
R value	0.5889	0.6355	0.6653	0.0719
P value	0.2188	**0.0146**	**0.0094**	0.7913
Deviation from zero?	Not Significant	Significant	Significant	Not Significant
**Total number of values**	6	14	14	16

Table 6: Linear regression (based on Pearson's correlation coefficient) between MAO-A/MAO-B variables: ***by diagnosis***

(i) Linear regression between MAO-A *activity* and MAO-B *activity*

Cortex

	Pooled (Male + Female)		
	**CTL**	**EOAD**	**LOAD**
**Slope**	5.900 ± 0.9530	7.529 ± 2.074	6.404 ± 2.896
**Goodness of Fit**			
R value	0.7842	0.6963	0.4837
P value	**< 0.0001**	**0.0027**	**0.0419**
Deviation from zero?	Significant	Significant	Significant
**Total number of values**	26	16	18

Hippocampus

	Pooled (Male + Female)		
	**CTL**	**EOAD**	**LOAD**
**Slope**	6.709 ± 1.699	5.212 ± 2.772	-1.496 ± 4.186
**Goodness of Fit**			
R value	0.7025	0.4624	0.0890
P value	**0.0012**	0.0827	0.7255
Deviation from zero?	Significant	Not Significant	Not Significant
**Total number of values**	18	15	18

(ii) Linear regression between MAO-A *protein* and MAO-B *protein*

Cortex

	Pooled (Male + Female)		
	**CTL**	**EOAD**	**LOAD**
**Slope**	0.1634 ± 0.4596	1.531 ± 0.7843	0.2748 ± 0.3932
**Goodness of Fit**			
R value	0.0724	0.4625	0.1721
P value	0.7253	0.0713	0.4947
Deviation from zero?	Not Significant	Not Significant	Not Significant
**Total number of values**	26	16	18

Hippocampus

	Pooled (Male + Female)		
	**CTL**	**EOAD**	**LOAD**
**Slope**	0.2962 ± 0.3345	-0.4304 ± 0.4617	-0.03646 ± 0.4557
**Goodness of Fit**			
R value	0.2229	0.2504	0.0200
P value	0.3899	0.3682	0.9372
Deviation from zero?	Not Significant	Not Significant	Not Significant
**Total number of values**	17	15	18

(iii) Linear regression between MAO-A *mRNA* and MAO-B *mRNA*

Cortex

	Pooled (Male + Female)		
	**CTL**	**EOAD**	**LOAD**
**Slope**	0.2637 ± 0.05625	0.2228 ± 0.2525	0.2299 ± 0.4478
**Goodness of Fit**			
R value	0.6913	0.2295	0.1273
P value	**< 0.0001**	0.3924	0.6147
Deviation from zero?	Significant	Not Significant	Not Significant
**Total number of values**	26	16	18

Hippocampus

	Pooled (Male + Female)		
	**CTL**	**EOAD**	**LOAD**
**Slope**	-0.3691 ± 0.2683	0.01698 ± 0.2307	0.6909 ± 0.3688
**Goodness of Fit**			
R value	0.3253	0.0204	0.4241
P value	0.1878	0.9424	0.0794
Deviation from zero?	Not Significant	Not Significant	Not Significant
**Total number of values**	18	15	18

Table 7: Linear regression (based on Pearson's correlation coefficient) between the activity of MAO isoforms and their respective proteins: ***by diagnosis***

(i) Linear regression between MAO-A *activity* and MAO-A *protein*

Cortex

	Pooled (Male + Female)		
	**CTL**	**EOAD**	**LOAD**
**Slope**	2.711 ± 7.425	5.778 ± 13.28	-11.47 ± 4.618
**Goodness of Fit**			
R value	0.0743	0.1155	0.5277
P value	0.7182	0.6701	**0.0244**
Deviation from zero?	Not Significant	Not Significant	Significant
**Total number of values**	26	16	18

Hippocampus

	Pooled (Male + Female)		
	**CTL**	**EOAD**	**LOAD**
**Slope**	24.08 ± 9.443	-6.570 ± 8.600	-0.8607 ± 7.143
**Goodness of Fit**			
R value	0.5375	0.2073	0.0301
P value	**0.0214**	0.4586	0.9056
Deviation from zero?	Significant	Not Significant	Not Significant
**Total number of values**	18	15	18

(ii) Linear regression between MAO-B *activity* and MAO-B *protein*

Cortex

	Pooled (Male + Female)		
	**CTL**	**EOAD**	**LOAD**
**Slope**	1.767 ± 24.81	76.92 ± 38.53	35.65 ± 44.18
**Goodness of Fit**			
R value	0.0145	0.4707	0.1977
P value	0.9438	0.0657	0.4316
Deviation from zero?	Not Significant	Not Significant	Not Significant
**Total number of values**	26	16	18

Hippocampus

	Pooled (Male + Female)		
	**CTL**	**EOAD**	**LOAD**
**Slope**	27.41 ± 76.42	8.718 ± 57.59	130.4 ± 57.27
**Goodness of Fit**			
R value	0.0922	0.0419	0.4947
P value	0.7248	0.882	**0.0369**
Deviation from zero?	Not Significant	Not Significant	Significant
**Total number of values**	17	15	18
